# Association between Farming and Chronic Energy Deficiency in Rural South India

**DOI:** 10.1371/journal.pone.0087423

**Published:** 2014-01-27

**Authors:** Asvini K. Subasinghe, Karen Z. Walker, Roger G. Evans, Velandai Srikanth, Simin Arabshahi, Kamakshi Kartik, Kartik Kalyanram, Amanda G. Thrift

**Affiliations:** 1 Department of Medicine, Monash Medical Centre, Southern Clinical School, Monash University, Melbourne, Victoria, Australia; 2 Department of Nutrition and Dietetics, Monash University, Melbourne, Victoria, Australia; 3 Department of Physiology, Monash University, Melbourne, Victoria, Australia; 4 Rishi Valley Rural Health Centre, Bangalore, Andhra Pradesh, India; 5 Florey Neuroscience Institutes, Melbourne, Victoria, Australia; Klinikum rechts der Isar der TU München, Germany

## Abstract

**Objective:**

To examine factors associated with chronic energy deficiency (CED) and anaemia in disadvantaged Indian adults who are mostly involved in subsistence farming.

**Design:**

A cross-sectional study in which we collected information on socio-demographic factors, physical activity, anthropometry, blood haemoglobin concentration, and daily household food intake. These data were used to calculate body mass index (BMI), basal metabolic rate (BMR), daily energy expenditure, and energy and nutrient intake. Multivariable backward stepwise logistic regression was used to assess socioeconomic and lifestyle factors associated with CED (defined as BMI<18 kg/m^2^) and anaemia.

**Setting:**

The study was conducted in 12 villages, in the Rishi Valley, Andhra Pradesh, India.

**Subjects:**

Individuals aged 18 years and above, residing in the 12 villages, were eligible to participate.

**Results:**

Data were available for 1178 individuals (45% male, median age 36 years (inter quartile range (IQR 27–50)). The prevalence of CED (38%) and anaemia (25%) was high. Farming was associated with CED in women (2.20, 95% CI: 1.39–3.49) and men (1.71, 95% CI: (1.06–2.74). Low income was also significantly associated with CED, while not completing high school was positively associated with anaemia. Median iron intake was high: 35.7 mg/day (IQR 26–46) in women and 43.4 mg/day (IQR 34–55) in men.

**Conclusions:**

Farming is an important risk factor associated with CED in this rural Indian population and low dietary iron is not the main cause of anaemia. Better farming practice may help to reduce CED in this population.

## Introduction

Chronic energy deficiency (CED) [1] and anaemia [2] are important manifestations of poor nutritional health. Both conditions are major problems in low to middle income countries with developing economies, including India; a country that has approximately 217 million (26%) of the World's undernourished population [3].

CED, the consequence of insufficient energy and protein intake, is defined in adults by a body mass index (BMI) <18 kg/m^2^ [4]. Reported prevalence rates of under-nutrition in India are as high as 60% in Madhya Pradesh and Orissa [5], with the problem particularly severe for pregnant Indian women [6], [7], children [8], and the elderly [9], [10].

Anaemia, as evidenced by low blood haemoglobin, is also a widespread problem in India [11]–[13] particularly for women of child-bearing age [14], children [15] and adolescent girls [15], [16]. In India anaemia is considered to be largely attributed to iron deficiency although paradoxically, Indian diets often provide high amounts of iron [12].

The occupation of farming may be another risk factor for CED and anaemia in rural India. The energy expenditure of farmers, particularly males from developing countries, has been found to range between moderate to high levels [17]. This can lead to the risk of underweight in these populations if adequate nutritional requirements are not met. In addition, farmers who wear exposed footwear or no shoes at all in the fields may be at risk of contracting infections. This is common practice in rural Indian populations [18]. However, there is a paucity of evidence for an association between farming and nutritional disorders in rural Indian populations.

In this study we have examined the impact of farming, and socioeconomic and dietary factors, on CED and anaemia in a rural south Indian community. We hypothesised that farming as an occupation would be associated with an increased risk of CED and anaemia.

## Methods

### Ethics statement

This study was conducted according to the guidelines laid down in the Declaration of Helsinki and all procedures involving human participants were approved by the Ethics Committees of Monash University, the Rishi Valley Education Centre and the Indian Council of Medical Research. Written informed consent was obtained by residents prior to any interviews or physical examinations.

### Study region and study population

This cross-sectional study was conducted in the north western region of the Chittoor District of Andhra Pradesh, India from 2006 to 2007. The residents are primarily subsistence farmers and earn an average monthly income for a family of five of 1,000 to 1,500 rupees ($0.80 to $1.25 per day). The survey was conducted in 12 villages surrounding the Rishi Valley Rural Health Centre.

#### Recruitment and baseline assessment

All adults residing in the 12 villages were eligible to participate. In collaboration with village leaders, public meetings were held in each village to inform residents about the study. Following this meeting, health workers visited the village and approached all villagers on a house to house basis. Informed consent was then obtained from those who presented at a local centre the next morning. All participants in the study underwent physical examinations to obtain measures of body height, weight, and hip and waist circumference. Haemoglobin levels were measured using a point of care device. Information on lifestyle and dietary intake was obtained from questionnaires. Details of the study and its methods have been described elsewhere [19].

### Questionnaires

Two questionnaires were administered to participants in the study: a demographic questionnaire and a household questionnaire. Both were translated into Telugu, the local language, and then back translated into English to identify and remove errors. All adult household members participating in the original study completed an 87-item demographic/lifestyle questionnaire administered by trained health workers. From this questionnaire, we obtained information on age, gender, occupation, income; and lifestyle factors including usual physical activity, alcohol consumption and smoking habits.

The individual responsible for the cooking in each household also completed a 55-item cooking/spending questionnaire. This questionnaire included questions about the portions of each food type and the amount of salt used daily in household cooking. Food types included vegetables, cereals, lentils, meat, fats and rice. Twelve standard cooking pots (5 millilitres to 1 litre volumes) were provided as visual aids to help the respondent estimate the amounts of each food type used when cooking. These vessels have been validated by the National Nutrition Monitoring Bureau (NNMB) of Andhra Pradesh.

Additionally, one of us (Dr Kamakshi Kartik), a Medical Practitioner from the Rishi Valley Rural Health Centre, used her knowledge of the local women and their usual cooking practices to provide a list of the most commonly consumed items within each food category. The main vegetables were onions, tomatoes, eggplant, okra, potatoes, peanuts, chilli, green beans, string beans, radish, tamarind, and amaranth. Meat and protein comprised poultry, eggs and mutton (goat meat). Lentils were categorised into green, red and chana dhal, plus flaxseed. The main cereal was rice, supplemented with millet and barley. The main fats were butter or ghee, plus sunflower, rapeseed, palm or groundnut oil. The main condiment used was chutney (varieties included tamarind, tomato and lentil).

### Physical measurements

Height was measured to the nearest 0.1 cm using a standard portable stadiometer (SECA 214, Hamburg, Germany). Weight was measured, in light clothes, to the nearest 0.1 kg using a set of standard calibrated portable electric scales placed on a hard-level surface (Soehnle, Germany). BMI was calculated by dividing body weight in kilograms by height in metres-squared. A finger-prick blood sample was taken to measure blood haemoglobin concentration using a HemoControl device (EKF-diagnostic, Magdeburg, Germany).

### Determination of energy expenditure

Basal metabolic rate (BMR) was calculated for each individual, based on BMI, using the age and gender-specific equations of Schofield [20]. Each BMR was multiplied by an activity factor of 1.8 (based on knowledge of the high physical activity levels usual for this population) in order to determine an individual's total energy expenditure (TEE) [20], [21]. Once the TEE for each individual in a household was calculated, the total energy requirement for the household as a whole was calculated by summation. The proportion of the total household energy requirement needed by each individual in the household was then expressed as a percentage of this total amount.

### Estimating food intake per household and per individual

Volumes of individual foods eaten by each household were estimated from the proportionate contribution that the food commonly made within its food group and the total volume of that food group consumed (based on the cooking/spending questionnaire). A volume conversion factor (taking into account volume change on cooking) was then applied to obtain food weight in grams. In cases where the entire food would not be eaten (eg: meat with bones), an edible portion factor specific to the food was also used. The energy, macronutrient, calcium and iron content of each food was then calculated using Indian Food Composition tables [22] or American Food Composition tables [23] when Indian data were unavailable.

As data on food consumption were collected by household and not by individual, energy and nutrient intake for individuals was estimated proportionately based upon the assumption that food would be distributed in the household according to individual energy requirements; i.e. individual nutrient intake  =  total household nutrient intake x proportion of household TEE required by that individual (see details under “Determination of energy expenditure” above). This approximated proportion method has been used in other studies [24], [25].

#### Missing data

Household questionnaires included dietary information for all people in the household. Sometimes people within these households did not complete the individual lifestyle questionnaire and/or undergo the physical examination. We therefore made some assumptions about these missing individuals pertaining to demographic and anthropometric information. This was necessary to calculate individual nutrient intakes from household dietary data. When age and gender, but no anthropometric data were available (n = 58), we assigned the mean height and weight of other individuals of the same age and gender to that individual. When neither age nor gender were available, but we knew from our census, or from other data, that the individual was an adult (n = 134), the individual was assumed to be a male aged 25 years since 25 year old males have the highest adult energy requirements.

One village, where fewer than 50% of households completed the cooking/spending questionnaires, was excluded from further analyses. Additionally, households with incomplete dietary data were excluded from the nutritional component of data analysis.

### Definitions

The World Bank classifies poverty as living on less than $1.25USD a day [26]. In our study, we defined a *low household income* when the household had an average monthly income of ≤1,000 rupees (approximately $19 AUD), and a ‘*not low’ income* as >1,000 rupees per month. *Educational attainment* was categorised as: no schooling, less than primary school, primary school, secondary school, high school, university or post graduate completed. *Work categories* assessed were: employed (non-government employee, government employee, and self-employed), non-paid, home-maker or unemployed (including students and retired individuals). Interviewers were instructed to tick the ‘non-paid’ option for participants engaged in farming or other work. Instead of sub-categorising farmers according to the type of agricultural work they performed, we categorised them according to whether or not they did farming work (this included agricultural labour, working as a cultivator or tenant cultivator). This category did not include individuals who owned land and did not work on it. The final work categories used, therefore, were non-government employee, government employee, self-employed, farming, homemaker, and unemployed.

Cut off values specific for Indian adults were used to categorise BMI into four categories. These values were prepared by a Consensus group in India after the WHO allowed revised guidelines to be implemented by each government in Asian countries [4].*Obesity*: BMI >25 kg/m^2^; *overweight*: BMI 23 kg/m^2^ to 24.9 kg/m*^2^, normal weight*: BMI 18 kg/m^2^ to 22.9 kg/m^2^; *underweight*: BMI <18 kg/m^2^. Grades of CED were defined as- Grade I: BMI 17 kg/m^2^ to 17.9 kg/m^2^; Grade II: BMI 16 kg/m^2^ to 16.9 kg/m^2^; Grade III: BMI <16 kg/m^2^. *Anaemia* was defined by the presence of a blood haemoglobin concentration <13 g/dL in men or <12 g/dL in non-pregnant women and <11 g/dL for pregnant women [27].

Individuals in the study population were also classified according to their traditional social status; either as *traditionally advantaged groups* or *traditionally marginalised groups. Traditionally advantaged groups* indicate those who are not eligible for ‘reservation benefits’ from the Indian Government. Traditionally, these individuals have professions in teaching or law and/or own land. *Traditionally marginalised groups* comprised individuals who are traditionally, financially, or educationally disadvantaged.

### Data analysis

Data were analysed using STATA (Version 11). As most variables were non-normally distributed, data are reported as medians and inter quartile ranges (IQRs). Chi-squared (χ^2^) tests were used to analyse categorical variables. A two-sided *P* value ≤0.05 was considered statistically significant.

Logistic regression was used to analyse the associations between exposure variables including demographic and dietary factors with the outcome variables CED and anaemia. Variables identified with a *P*>0.1 were then removed using backward stepwise multivariable logistic regression to determine variables independently associated with CED and anaemia. Variables were removed one at a time, apart from age, until all remaining variables in the model had a *P* value <0.1. Collinearity between variables was evaluated using least squares regression. Participants with missing data were excluded before these analyses were undertaken.

## Results

A total of 1479 adults responded to the lifestyle questionnaire in the original study. Of these, 1178 individuals belonged to households in which dietary questionnaires had been completed, from 11 of the 12 villages, and therefore were included in this present analysis. The median age for this sample was 36 (IQR 27-50) years. Approximately half (45%) were male. Although an additional 192 adults were known from the census to be living within the households surveyed, anthropometric data and sometimes age or gender were unavailable for these people. There was no major collinearity between the independent variables income, education, socioeconomic advantage, and work category (variance inflation factor (VIF) <5). Demographic features of participants who responded to the lifestyle questionnaire and who belonged to households in which dietary information was collected are presented in Table 1.

**Table 1:** Demographic factors of the study population.

Demographic Factors	Women (n = 648)		Men (n = 530)	
	N	%	N	%
**Age (years)**				
18–24	112	17.3	89	16.8
25–34	188	29.0	137	25.9
35–44	146	22.5	114	21.5
45–54	94	14.5	85	16.0
≥55	108	16.7	105	19.8
**BMI (kg/m^2^)**				
18–22.9	310	47.8	269	50.8
<17	176	27.2	103	19.4
≥17–17.9	93	14.4	78	14.7
≥22.9–24.9	35	5.4	48	9.1
>25	34	5.3	32	6.0
Traditional social status*				
Traditionally Advantaged Groups	201	31.3	140	26.6
Traditionally Marginalised Groups	441	68.7	387	73.4
**Highest education level**†				
No schooling	421	65.6	170	32.2
Less than Primary school	50	7.8	84	15.9
Primary school	35	5.5	44	8.3
Secondary school	59	9.2	65	12.3
High school	69	10.7	123	23.3
University	5	0.8	30	5.7
Postgraduate degree	3	0.5	12	2.3
Work Category*				
Non Govt/Govt employee	170	26.5	206	39.1
Self-employed	65	10.1	100	19.0
Farming and livestock	156	24.3	170	32.3
Homemaker	208	32.4	1	0.2
Unemployed/Student/Retired	43	6.7	50	9.5
**Monthly household income level (rupees)**‡				
≤1,000	409	63.9	273	51.7
1,001 to 2,000	148	23.1	161	30.5
2,001 to 3,000	48	7.5	49	9.3
3,001 to 4,000	10	1.6	15	2.8
>4000	20	3.1	26	4.9
Refused	5	0.8	4	0.8
**Iron intake (mg/day)**				
Meeting RDA	573	88.4	510	96.2
Below RDA	75	11.6	20	3.8
**Energy intake (mg/day)**				
Meeting RDA	589	90.9	489	92.3
Below RDA	59	9.1	41	7.7
Anaemia§**				
Yes	213	34.2	78	14.8
No	410	65.8	449	85.2

BMI, Body Mass Index; RDA,Recommended Daily Allowance; Govt, Government. * Assessed in 642 women,527 men; † Assessed in 642 women, 528 men; ‡ Assessed in 640 women, 528 men;§ Assessed in 623 women,527 men;**Anaemia was defined as a blood haemoglobin concentration <13 g/dL in men or <12 g/dL in non-pregnant women and <11 g/dL for pregnant women. RDA, Recommended Daily Allowance; Govt, Government

Approximately 28% of individuals were engaged in farming work (Table 1). There were similarities between men and women with respect to socioeconomic indicators. Most men and women in the study population were from *traditionally marginalised groups* (73% men, 69% women; *P* = 0.076) and were in the lowest income and education categories. More men (58%) than women (37%) were employed (*P*<0.001). Women (32%) were more likely than men (0.2%) to be homemakers (*P*<0.001) and to never have attended school (66% women, 32% men; *P*<0.001).

**Figure 1:**
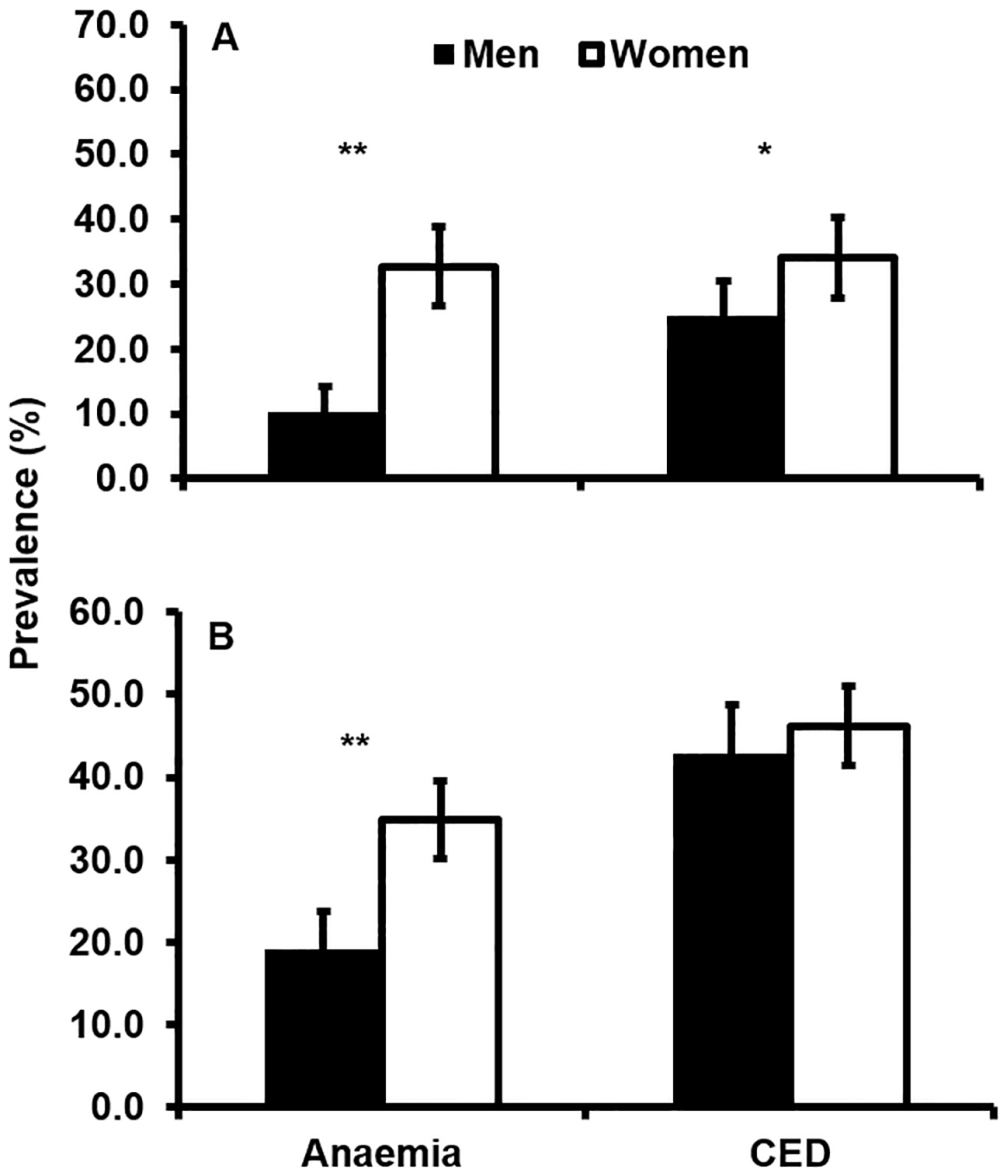
Prevalence of Anaemia and CED in men and women aged 18 years and over. Panel 1A shows data for ‘not low’ income families (>1,000 rupees per month) while panel 1B shows data for low income families (≤1,000 rupees per month). Anaemia was defined according to WHO individual haemoglobin (Hb) levels: Hb <12 g/dL for non-pregnant women, Hb <11 g/dL for pregnant women, and Hb <13 g/dL for men. CED was defined in both genders as a BMI <18 kg/m^2^. (n = 524 men and 635 women). Error bars show 95% Confidence Intervals. Black bars show data for men and white bars show data for women. ** denotes significance at p<0.001;* denotes significance at p<0.05.

Adults from low income families appeared to be more likely to have anaemia (*P* = 0.003) and CED (*P*<0.001) than those in ‘not low’ income families. Anaemia was more prevalent in women than men (Figure 1A and 1B). CED was more prevalent in women than men in higher income families, and in families with a low income (Figure 1 A and 1B).

Women who were farmers had CED (52%) more often than women who were homemakers or non-government/government employees (p<0.05) (Table 2). Women from low income families had CED (46%) more often than women from higher income families (34%; p<0.01). Similar associations were found in men.

**Table 2:** Prevalence of chronic energy deficiency according to socio demographic factors.

	Body mass index <18 kg/m^2^	
Socio-demographic factor	Women n* (%)	Men n* (%)
**Age**		
18–24	48 (43)	45 (51)
25–34	88 (47)	47 (34)
35–44	59 (40)	28 (25)
45–54	31 (33)	19 (22)
≥55	43 (40)	42 (40)
**Household monthly income**		
Not low income (>1000rupees)	77 (34)	63 (25)
Low income (≤1000 rupees)	189 (46)	117 (43)
**Work category**		
Non Govt/Govt employee	64 (38)	56 (27)
Self-employed	29 (45)	26 (26)
Farming and livestock	81 (52)	70 (42)
Homemaker	79 (38)	0 (−)
Unemployed/Student/Retired	15 (35)	27 (54)

BMI, Body Mass Index; Govt, Government. *A total of 269 women and 181 men had a BMI<18 kg/m^2^

**Figure 2:**
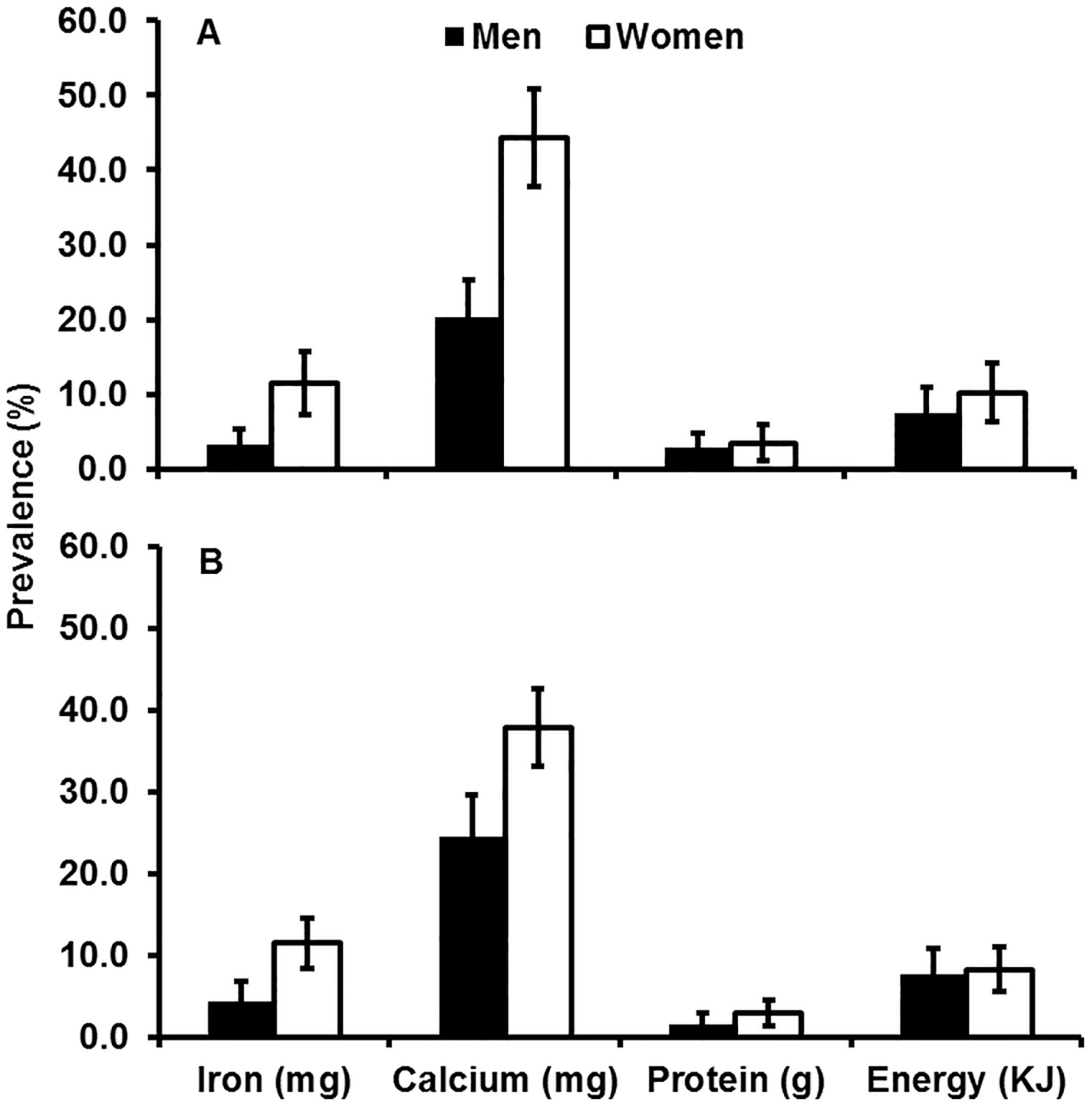
Prevalence of daily nutrient and energy intake deficiencies in men and women aged 18 years and over. Panel 2A shows data for ‘not low’ income families (>1,000 rupees per month) while panel 2B shows data for low income families (≤1,000 rupees per month). (n = 524 men and 635 women). Error bars show 95% Confidence Intervals. Black bars show data for men and white bars show data for women.

In adults from low and higher income families, the most common micronutrient deficiency was calcium, while, deficiencies in iron, protein and energy were relatively infrequent (Figure 2A and 2B). However, women were more deficient in iron than men in both low income (*P* = 0.001) and ‘not low’ income families (*P*<0.001).

### Risk factors for CED and Anaemia

#### CED

In multivariable analysis, older age was independently negatively associated with CED in both men and women (45–55 years; Table 3). Men (OR 1.71, 95% CI 1.06–2.74, *P* = 0.026) and women (OR 2.20, 95% CI 1.39–3.49, *P* = 0.001) who engaged in farming had a greater risk of having CED compared with those engaged in other paid employment. Additionally, independent of age, unemployed men appeared to have a greater risk of CED (OR 2.41, 95% CI 1.17–4.97, *P* = 0.017) than other men. Independent of employment status, men with a lower family income were at greater risk of CED (OR 2.03, 95% CI 1.33–3.11, *P* = 0.001). Women from traditionally advantaged groups had a lower risk of having CED (OR 0.67, 95% CI 0.46–0.97, *P* = 0.035) than women from traditionally marginalised groups. Farming was associated with CED independently of income. Importantly, we found no significant relationship between low energy intake and the risk of CED in either men or women.

**Table 3:** Univariable and multivariable* logistic regression analyses of factors associated with chronic energy deficiency (body mass index <18 kg/m^2^) in women and men aged ≥18 years.

	Women				Men			
Variables	Univariable		Multivariable		Univariable		Multivariable	
	OR (95% CI)	*P* value	OR (95% CI)	*P* value	OR (95% CI)	*P* value	OR (95% CI)	*P* value
**Age (years)**								
18–24	1.0		1.0		**1.0**		**1.0**	
25–34	1.17 (0.73–1.88)	0.506	1.06 (0.64–1.74)	0.824	**0.51 (0.30–0.88)**	**0.016**	**0.53 (0.29–0.96)**	**0.035**
35–44	0.90 (0.55–1.49)	0.693	0.81 (0.48–1.36)	0.424	**0.32 (0.18–0.58)**	**<0.001**	**0.28 (0.15–0.54)**	**<0.001**
45–54	0.66 (0.37–1.16)	0.147	**0.54 (0.30–0.99)**	**0.047**	**0.28 (0.15–0.54)**	**<0.001**	**0.22 (0.11–0.45)**	**<0.001**
≥55	0.88 (0.52–1.51)	0.647	0.80 (0.45–1.44)	0.460	0.65 (0.37–1.15)	0.141	**0.35 (0.18–0.68)**	**0.002**
**High School**								
Completed	1.0		–		1.0		1.0	
Not Completed	1.57 (0.95–2.60)	0.080	–		**1.51 (1.01–2.26)**	**0.043**	1.58 (0.97–2.60)	0.068
**Household Monthly Income (rupees)**								
>1000	1.0		1.0		1.0		1.0	
≤1000	**1.66 (1.19–2.33)**	**0.003**	**1.68 (1.17–2.41)**	**0.005**	**2.24 (1.54–3.25)**	**<0.001**	**2.03 (1.33–3.11)**	**0.001**
**Traditionally advantaged group**	**0.62 (0.44–0.87)**	**0.006**	**0.67 (0.46–0.97)**	**0.035**	**0.53 (0.34–0.82)**	**0.004**	–	
**Work**								
Non Govt/Govt employed	1.0		1.0		1.0		1.0	
Self-employed	1.33 (0.75–2.38)	0.329	1.42 (0.78–2.57)	0.250	0.94 (0.55–1.62)	0.826	0.85 (0.48–1.50)	0.567
Farming and livestock	**1.79 (1.15–2.78)**	**0.010**	**2.20 (1.39–3.49)**	**0.001**	**1.87 (1.22–2.89)**	**0.004**	**1.71 (1.06–2.74)**	**0.026**
Homemaker	1.01 (0.67–1.54)	0.947	1.14 (0.74–1.76)	0.557	–		–	
Unemployed†	0.89 (0.44–1.79)	0.738	0.88 (0.40–1.94)	0.747	**3.14 (1.67–5.93)**	**<0.001**	**2.41 (1.17–4.97)**	**0.017**
**Energy (kJ/day)‡**								
Meeting RDA	1.0				1.0		–	
Below RDA	1.04 (0.60–1.79)	0.888			1.26 (0.65–2.42)	0.494	–	

Govt, Government; RDA, Recommended Daily Allowance.*Multivariable analyses were adjusted for all other variables in the column. The multivariable analysis for women included 635 observations and the pseudo-*R* ^2^ was 0.0383 and for men there were 522 observations and the pseudo-*R^2^* was 0.0844. Value*s* in bold are significant at a *P*≤0.05. †Unemployed include students and retirees. ‡Low energy intake for women was defined as <9330 kJ/day and <11422 kJ/day for men.

#### Anaemia

In multivariable analysis, greater age was independently associated with a higher risk of anaemia in women (35–44 years) (OR 2.17, 95% CI 1.22–3.86, *P* = 0.008). Conversely, men (OR 0.17, 95% CI 0.02–1.26, *P* = 0.083) and women (OR 0.27 95% CI 0.09–0.81, *P* = 0.019) who were overweight had a lower risk of having anaemia compared with those who had a BMI in the normal range. Women who engaged in farming (OR 1.56, 95% CI 0.97–2.51, *P* = 0.068) had a greater risk of anaemia compared to women who were in the non-government/government employment category (Table 4). Men who did not complete high school (OR 2.26, 95% CI 1.09–4.67, *P* = 0.028) were more likely to be anaemic than men who did not. Additionally, we found no significant relationship between poor iron intake and the risk of anaemia in either men or women.

**Table 4:** Univariable and multivariable logistic regression analyses of factors associated with anaemia* in women and men aged ≥18 years.

	Women				Men			
Variables	Univariable		Multivariable		Univariable		Multivariable	
	OR (95% CI)	*P* value	OR (95% CI)	*P* value	OR (95% CI)	*P* value	(95% CI)	*P* value
**Age (years)**								
18–24	1.0		1.0		1.0		1.0	
25–34	1.64 (0.96–2.81)	0.070	1.65 (0.95–2.85)	0.074	0.60 (0.25–1.46)	0.262	0.69 (0.28–1.69)	0.411
35–44	**1.94 (1.11**–**3.38)**	**0.019**	**2.17 (1.22**–**3.86)**	**0.008**	1.05 (0.45–2.41)	0.914	1.04 (0.44–2.49)	0.928
45–54	1.39 (0.74–2.59)	0.308	1.44 (0.76–2.76)	0.264	1.14 (0.47–2.74)	0.777	1.12 (0.44–2.88)	0.805
≥55	1.59 (0.87–2.92)	0.131	1.62 (0.85–3.09)	0.142	**2.67 (1.24**–**5.73)**	**0.012**	2.10 (0.92–4.78)	0.076
**Body mass index (kg/m^2^)**								
18–22.9	1.0		1.0		1.0		1.0	
<17	1.28 (0.87–1.89)	0.213	1.27 (0.85–1.88)	0.238	**1.98 (1.11**–**3.53)**	**0.020**	**1.91 (0.99**–**3.36)**	**0.033**
17–17.9	1.02 (0.63–1.68)	0.918	0.95 (0.57–1.57)	0.836	1.31 (0.66–2.61)	0.446	1.29 (0.63–2.66)	0.487
23–24.9	**0.26 (0.09**–**0.77)**	**0.014**	**0.27 (0.09**–**0.81)**	**0.019**	0.14 (0.02–1.02)	0.053	0.17 (0.02–1.26)	0.083
>25	0.42 (0.17–1.05)	0.064	**0.39 (0.15**–**0.98)**	**0.044**	0.92 (0.30–2.78)	0.883	1.0 (0.32–3.12)	0.997
**High School**								
Completed	1.0				1.0		1.0	
Not Completed	**1.87 (1.06**–**3.30)**	**0.032**	–		**3.15 (1.62**–**6.13)**	**0.001**	**2.26 (1.09**–**4.67)**	**0.028**
**Household Monthly Income (rupees)**								
>1,000	1.0				1.0			
≤1,000	1.10 (0.78–1.57)	0.580	–		**2.05 (1.23**–**3.39)**	**0.006**		
**Traditionally advantaged group**	0.82 (0.57–1.18)	0.293	–		1.03 (0.60–1.78)	0.914	–	
**Work**								
Non Govt/Govt employed	1.0		1.0		1.0		–	
Self-employed	1.36 (0.74–2.48)	0.320	1.27 (0.69–2.35)	0.443	1.50 (0.77–2.93)	0.234	–	
Farming and livestock	**1.60 (1.0**–**2.54)**	**0.047**	1.56 (0.97–2.51)	0.068	1.37 (0.76–2.46)	0.296	–	
Homemaker	1.06 (0.68–1.65)	0.806	1.11 (0.70–1.76)	0.647	–		–	
Unemployed†	1.34 (0.65–2.74)	0.425	1.40 (0.62–3.17)	0.416	1.59 (0.69–3.66)	0.276	–	
**Iron (mg/day)**‡			–					
Meeting RDA	1.0				1.0		–	
Below RDA	0.81 (0.48–1.38)	0.438			2.59 (0.96–6.96)	0.059		
**Energy (kJ/day)**§								
Meeting RDA	1.0		–		1.0		–	
Below RDA	1.04 (0.59–1.85)	0.881	–		1.24 (0.53–2.92)	0.618		

Govt, Government; RDA, Recommended Daily Allowance.*Anaemia was defined as a blood haemoglobin concentration <13 g/dL in men or <12 g/dL in non-pregnant women and <11 g/dL for pregnant women. Multivariable analyses were adjusted for all other variables in the column. The multivariable analysis included 622 observations and the pseudo-*R* ^2^ was 0.0332 for women and for men there were 527 observations and the pseudo-*R* ^2^ was 0.0833. Values in bold are significant at *P*≤0.05. †Unemployed include students and retirees. ‡Low iron intake was defined as <21 mg/day for women and < 17 mg/day for men. §Low energy intake was defined as <9330 kJ/day for women and <11422 kJ/day for men.

## Discussion

The novel finding from this study was that the occupation of farming significantly increased the risk of CED in disadvantaged men and women. In women, farming also increased the risk of anaemia. We found no significant associations between poor iron and energy intake with the risk of anaemia and CED. Instead, socioeconomic disadvantage was positively associated with an increased risk of anaemia and CED across both sexes. Importantly, the association between socioeconomic disadvantage and CED was independent of income. These observations suggest that while optimising diet is of obvious importance for overall health, interventions that focus solely on diet may have limited efficacy in reducing the prevalence of anaemia and CED in these populations.

We found that approximately 38% of our study population had CED. The mean BMI estimates and prevalence of CED we found in our study are consistent with estimates reported in other Indian populations (Tables 5 and 6). Few other studies have been undertaken to assess the relationship between farming and CED. One cross-sectional study conducted during drought in 1347 rural Indian adults provided evidence for a higher prevalence of CED in individuals at socioeconomic disadvantage and in households engaged in marginal and small sized farming [28]. In a survey, based on data from the 2005/06 National Nutrition Survey across twenty major Indian states, improved agricultural performance was found to markedly reduce the risk of CED in adults and body wasting in young children [29].

**Table 5:** Mean Body mass index (BMI) estimates from other Indian studies.

			Mean BMI (kg/m^2^)	
Study Population	Year	Sample size (n)	Women	Men
Rishi Valley (current study)	2006–2007	1178	19.0	19.4
Rajasthan, India [33]	1994	3148	21.1	21.4
Raipur Rani, Haryana, India [34]	1999	2559	19.1	18.7
Moradabad, India [35]	1997	566	20.2	20.6
Tamil Nadu, India [36]	2011	10, 463	21.9	21.4

**Table 6:** Prevalence of chronic energy deficiency (CED) in our study compared to in other Indian populations [37].

			Prevalence of CED (%)	
Study Population	Year	Sample size (n)	Women	Men
Rishi Valley (current study)	2006–2007	1178	42	34
Kerala	2002	2815	19	22
Madhya Pradesh	2002	3317	42	43
West Bengal	2002	2993	46	40
Gujarat	2002	2759	33	37

We found that unemployment was positively associated with the risk of CED in men, independent of income. Indeed the prevalence of CED in men who were unemployed was at least twice that in any other category of employment. This is consistent with data from a study of 469 adult males in Kolkata, where the prevalence of CED was greatest (40.5%) in households with an average monthly family income of less than 2,000 rupees [30]. Moreover risk decreased in those with monthly household incomes greater than 2,000 rupees [30].

Approximately 25% of adults in our study population were anaemic. This prevalence at a community level is considered by WHO as a problem of high magnitude [31]. Nevertheless, the dietary intake of iron for a large proportion of the study population appeared to be adequate. No relationship was evident between dietary iron intake and the prevalence of anaemia. Although it is possible that iron consumption in this population has been overestimated, these results may also point to the importance of factors such as poor iron absorption secondary to gastrointestinal infection or chronic blood loss [32].

We found that farming was strongly associated with CED in men and women, and was weakly associated with anaemia in women. Since most farm holdings are small (<0.5 acres) in this study population, the introduction of mechanised farming methods would be futile. However, if hookworm is a problem, then wearing enclosed footwear in the fields may help reduce contact with the soil and such exposure to infectious and parasitic organisms.

There were some limitations to the study as collecting data in rural populations is a challenge. Firstly, we excluded data from one village where >50% of households had not completed the dietary questionnaire. Among the remaining villages, although the response rate for individual questionnaires was reasonably high (71%), many of these respondents did not complete a household dietary questionnaire and so we had complete dietary data only for 80% of the 1479 surveyed. However, when we added individuals into the multivariable analysis who did not have dietary data, there were no significant differences in the associations we found (data not shown). Additionally, we did not measure dietary intake on a 24 hour recall basis, so we were unable to obtain precise estimates of energy received by individuals from their household food. Instead, we relied on estimations of individual food intake from total household consumption assuming that only people living in that household ate this food.

A further limitation is that we categorised occupations based on which role individuals considered as their major occupation in the last 12 months. This is because we were primarily interested in the level of activity associated with their main occupation. Similarly, we did not differentiate between types of agricultural labour in individuals who indicated that farming was their major occupation. This was because we were interested in the level of exposure individuals had with the fields, as opposed to what type of agricultural labour they were involved in.

Conversely, there were a number of strengths to this study. The study included data from a large sample size (*n* = 1178). Therefore, there was sufficient power to allow detection of effects of socioeconomic factors and dietary factors on nutritional status. We also offer novel evidence that farming plays a role in the risk of CED and anaemia. Our findings also suggest that, irrespective of adequate iron intake, anaemia in the population may relate to some circumstances of farming. These may include increased exposure to gastrointestinal diseases and parasite infections such as hookworm. However, as we did not measure this, we cannot provide direct support for this conclusion.

## Conclusion

In the present study, we found that CED and anaemia in the setting of Indian rural communities were associated with socioeconomic disadvantage and farming. For these communities, education on optimal farming and nutrition practices may therefore be important measures to promote good health.
